# Fictional Film in Engineering Ethics Education: With Miyazaki’s *The Wind Rises* as Exemplar

**DOI:** 10.1007/s11948-022-00399-w

**Published:** 2022-09-13

**Authors:** Sarah Jayne Hitt, Thomas Taro Lennerfors

**Affiliations:** 1 SFHEA, New Model Institute for Technology and Engineering, Blackfriars Street, Hereford, HR4 9HS UK; 2 Department of Civil and Industrial Engineering, Division of Industrial Engineering and Management, Uppsala University, Box 169, 751 04 Uppsala, Sweden

**Keywords:** Cinema, Pedagogy, Ethics, Engineering, Narrative, Arts-based methods

## Abstract

This paper aims to call attention to the potential of using film in engineering ethics education, which has not been thoroughly discussed as a pedagogical method in this field. A review of current approaches to teaching engineering ethics reveals that there are both learning outcomes that need more attention as well as additional pedagogical methods that could be adopted. Scholarship on teaching with film indicates that film can produce ethical experiences that go beyond those produced by both conventional methods of teaching engineering ethics and more arts-based methods such as fiction, as well as connect ethics learning outcomes and issues to the lifeworld of a person. The paper further illustrates the potential of using Miyazaki Hayao’s film *The Wind Rises* for highlighting a range of ethical issues pertaining to engineering. It also discusses the important role educators play in how film can be used effectively in the classroom. Synthesizing a range of sources from film theory to the use of film in business and medical education, the paper makes the case for using film in engineering ethics education and calls for more research on the use of this method.

## Introduction

Teaching with film has been of pedagogical interest since at least the 1970s, when Wegner (1977) developed a guide for the United States National Institute of Education describing the possibilities for using different types of films in the classroom, and advocating for incorporating film into learning environments. Within business ethics and medical ethics education, film is used to help achieve particular outcomes ranging from promoting critical thinking, preparing professionals for emotional aspects of their future work, cultivating virtues, and considering stakeholder perspectives (Searight & Allmayer, 2014; Skorin-Kapov & Benson, 2018; Tone Hosmer & Steneck, 1989). While the search terms “business ethics” AND “film” returns 17 papers and “medical ethics” AND “film” returns 170 papers on Scopus, when searching using the keywords “engineering ethics” AND “film,” there were only three papers that were returned, and only one of them namely Berne (2001) concerned the use of a fictional film, *Matrix*, to support learning in engineering ethics. In Hess and Fore’s (2018) systematic review of engineering ethics pedagogies in the United States, film was not mentioned.

Searching beyond Scopus rendered more papers, but all of them only mention the use of film in passing, e.g. to support a Science and Technology Studies inspired engineering ethics course (Riley, 2008), to highlight Kohlberg’s stages of moral development (Dyrud, 2001), as a way to critically re-examine love and relationships (van Grunsven, 2021), as a part of case study methodology (Martin et al., 2021), and to promote a phenomenological perspective (Troesch, 2015). Film is also mentioned in several other papers but given that the papers do not concern teaching with film, it is not well explained how it is used in engineering ethics education (van Grunsven et al., 2021; Berry et al., 2013, Clancy 2021). In addition, Riley (2013) from the perspective of feminist ethics, critiques the educational film *Henry’s Daughters* for representing women in a stereotypical way. In other words, educators and educational researchers within engineering ethics education are becoming aware of the potential of using film, and are beginning to try using it, but there has not been a rigorous examination of this pedagogy and its associated methodologies in engineering ethics education. Why, when film has been shown to be effective in business and medical education, is there such a lack of scholarship on the use of film in engineering ethics education?

A reason for this lack of publications focusing on film in engineering ethics could be that despite the extensive impact of engineering on daily life, we do not find many fictional films with engineers as protagonists or with engineering as an explicit theme, whereas many films and television series feature professionals in their work environments, for example nurses (Stanley, 2008), librarians (Shaffer & Casey, 2013), PR professionals (Ames, 2010), and scientists (Flicker, 2003; Kirby, 2010; Weingart et al., 2003). Nevertheless, we argue that because engineering ethics problems are present in films even where engineers themselves are not necessarily featured, engineering ethics educators might therefore adopt the approach of business ethics and medical ethics educators who have been using film in their teaching for several decades.

While engineers and engineering may not be prominently featured in popular films, some educational films have been deliberately produced as a tool for teaching engineering ethics, for example *Henry’s Daughters*, *Incident at Morales*, *Testing Water…and Ethics*, and *Ethicana*. Given that these films are designed for classroom use, they go deeper into the engineering ethics content. However, their quality and the emotional engagement that they arouse, although very good for its purpose, do not come close to that of fictional films. Indeed, as early as 1977, Wegner pronounced educational films like these a failure: “students detect the educational film almost immediately, for it is usually characterized by heavy-handed didactics and a notable lack of production value... [these films] are often clumsy and off-putting” (p. 10). Our purpose is not to denigrate these films, and there is evidence that these educational videos are effective at achieving engineering ethics learning outcomes (Itani, 2013; Loui, 2013).

Aside of these examples, though, film has not been sufficiently addressed in scholarship as a means for engineering ethics education. Because we will argue that watching film evokes emotions, imagination, and a connection to personal lived experiences, we believe that it can play a crucial role in this field, and help to achieve existing learning outcomes as well as to broaden these. At a time when more than ever, the world needs engineers who are compassionate, curious, dedicated to serving the public and to sustainable practices that positively impact the earth, engineering educators must use as many tools as possible to cultivate moral awareness and sensitivity and to inculcate the skills of ethical analysis and decision-making. For these reasons, teaching with film should be situated within the emerging literature of best practices in engineering ethics education.

The purpose of our paper is to make the case for using fictional film in engineering ethics education, and our argument unfolds as follows. We first give an overview of the key learning outcomes in engineering ethics education, and identify a disconnect between the desired learning outcomes and pedagogical practices, which often support an individualist, analytical, and cognitive approach, neglecting experiential and emotional learning. This provides an argument for the need for teaching methods such as film. We then describe how ethics has been discussed in relation to film, where we distinguish between a number of different connections, finally settling on one which argues that film can produce ethical experiences that viewers can then reflect upon. We then propose the use of Miyazaki’s film *The Wind Rises* in engineering ethics education. This is followed by a discussion about how educators who use film could achieve established learning outcomes as well as broaden them, and finally the potential of film beyond the engineering ethics classroom.

## Engineering Ethics Education

Before discussing the possible uses of film in engineering ethics education, it is necessary to describe the field’s aims and contexts so as to demonstrate where film can help fill existing gaps. Herkert (2000) argued that there is general agreement about the importance of engineering ethics education in increasing student sensitivity to ethical issues, increasing student knowledge of relevant standards, improving ethical judgment, and increasing ethical will-power. Pfatteicher (2001) takes a different position, arguing that students should understand the nature of engineering ethics in order to consider why one should be an ethical engineer, and be trained in resolving problems. Lennerfors et al. (2020) discuss awareness, responsibility, critical thinking, and action as possible learning outcomes in engineering ethics education, while Tormey et al. (2015) draw on James Rest’s four learning outcomes of moral sensitivity, moral judgment, moral motivation, and moral character.

While most of the researchers mentioned above agree that emotion, awareness, and moral sensitivity are important learning outcomes, there is critique that engineering ethics education has often ignored these, instead focusing narrowly on professional codes of ethics and workplace dilemmas (Walling, 2015). This focus has engendered a pedagogy that results in an individualist, analytical, cognitive approach to ethics [Mitcham (2009); for similar arguments, see Sunderland et al. (2014), Roeser (2012), Newberry (2004)], where a variety of frameworks are applied as an intellectual exercise, rarely translating into ethical action and commitment (Walling, 2015). This line of argument has also revealed the need for a more inclusive set of theories studied (including ethics of care, feminist ethics, pragmatism) and non-Western (e.g., Confucian) ethical theories (Haws, 2001; Jing & Doorn, 2020; Zhu & Jesiek, 2017), not least because of the global nature of engineering work (Chang & Wang, 2011; Lohmann et al., 2006; Murphy et al., 2015).

We agree that codes as well as moral reasoning are necessary in engineering ethics education, but that this should not be the only content in such education. A number of authors have presented suggestions for how to go in other directions. Sunderland (2019) argues for a student-centered approach to teaching ethics. Voss (2013) shows that students can learn engineering ethics more effectively through immersive personal and technological experiences. Mitcham (2009) and others argue that engineering should be made part of the lifeworld, subordinating ‘‘to efforts to advance a deeper human understanding of the Good’’ (p. 50). Morrison (2020) who is arguing for a postphenomenological approach, suggests that we should aim to include “the many small day-to-day decisions that frame the ethical conflicts that we may have to grapple with in the future” (p. 1392). Snieder and Zhu (2020) believe that because adolescents are naturally concerned with meaningful questions about their purpose and place in the world, professional ethics education should “connect to the heart” (p. 2244), situating engineers first and foremost as *humans* who are engaging with the lifelong development of their ethical values rather than simply as professional figures fulfilling a role (Snieder & Zhu, 2020).

To sum up, the current state of the engineering ethics education research debate is that although educators seemingly agree upon a number of important learning outcomes, there is a critique that such education in practice is narrowly construed. We are inspired by the opportunities that film pedagogy offers to broaden engineering ethics education practice but also to create more impactful learning experiences for students.

## Ethics and Film

Robert Sinnerbrink, who at depth has explored the relationship between ethics and film, argues that alongside the development of cinema there has always been a discussion about its ethical effects. In summarizing the previous scholarly discussion on the emerging theory of the relationship between ethics and film, Sinnerbrink separates it into three different strands. *Ethics in cinema* focuses on the narrative content of the film, including its story and the various ethical situations that the protagonists find themselves in. *Ethics of cinematic representation* focuses on the ethics of film production and reception, for example discussing whether documentaries constitute truthful accounts of reality. *Ethics of cinema as a medium expressing moral beliefs and ideology*, comprises the ideological underpinnings of movies from critical perspectives, such as Marxism, feminism, and ecocritical perspectives. Sinnerbrink’s own view is that what is lacking from the discussion is a consideration of *cinema as a medium of ethical experience*. He highlights the transformative potential of cinema to sharpen our ethical perceptions and to provide experience of moral-social complexity. He connects this to the aesthetics of cinema, since its particular aesthetics is a key to bringing forth ethical reflection in different ways than is possible through other media or artistic modalities. Cinematic ethics involves “an education of our senses and exercise of our moral imaginations that require not only an acknowledgment of, or responsiveness to, ethical concerns but careful attention to aesthetic composition and sensitivity to cinematic technique.” (Sinnerbrink, 2019, p. 199).

Sinnerbrink argues that film can lead to ethical experience in three related ways. First, the ethical experience is elicited by the shared cinematic experience of engaging with fictional characters’ actions, reflections, perspectives, and experiences as they are represented in interpersonal, social, and political situations. Through an emotional connection to the fictional characters, the complexity of ethical action is highlighted. The embeddedness and narrative structure of the characters’ ethical reflection shows that “moral rules and concepts are too thin to determine the particular situations that fall under them”. Rather, moral judgment is required, “and the capacity for moral judgment is exactly what is ideally exercised and refined through our encounters with narrative artworks” (Carroll, 1998, p. 146). Film, then, allows for what we would like to call “thick” contexts to be presented, enabling reflection on the kind of judgments and actions that are made in response to complicated situations. The fictional characters also become exemplars of ethical conduct, which the viewers could identify with and learn from. But apart from the identification or disidentification between the viewer and the fictional characters, the viewers, secondly, are invited to reflect on their feelings, emotional engagement, or moral sympathy or antipathy. This allows the viewers to distance themselves from the film and reflect upon what the characters did right or wrong, or how they should have acted if they were in the same situation as the fictional character. Third, cinematic experiences are brought about by aesthetic means which can involve broadening the viewers’ ethical horizon. Crucial is Sinnerbrink’s (2018) take that “spectators may reach a deeper understanding of moral situations by having their assumptions or beliefs challenged via complex forms of emotional engagement coupled with critical reflection arising from this ethical experience” (p. 199).

We have presented this scholarship on the relationship between ethics and film to provide a theoretical grounding for our proposal that engineering educators adopt film as a medium for teaching ethics. While a firm grounding in film theory is not necessary, educators should however be cognizant of the current conversation regarding investigations into the possibilities that film provides for explorations of philosophical and ethical concerns in the classroom. In addition, scholars within professional ethics have shown that the use of film discussion is useful in promoting positive change in students’ moral reasoning skills (Green et al., 1995; Self et al., 1993), in priming our imagination (Carson, 1994), in fostering experiential learning and critical thinking (Bluestone, 2000), and in developing moral knowledge and awareness (Villalba & Redmond, 2008). So, despite the lack of scholarship on the use of film in engineering ethics education specifically, we argue that the available resources on teaching ethics through film and the burgeoning research on teaching engineering ethics with speculative fiction may be synthesized to elicit some pedagogies which have great potential. Our own take (which we have developed through our own experience as viewers as well as through our experience in using film in the engineering ethics classroom) is that there are several reasons why film can be used for teaching engineering ethics.

## Film in Engineering Ethics Education

The purpose of fictional film is not necessarily to engender a moral reaction from viewers or to produce moral action. Yet of course, many films do. Scholars attribute this reaction to the link between narrative and ethics (Carroll, 1998; Nussbaum, 1990). Indeed, a foundational concept in the field of narrative ethics is the understanding that “narratives themselves implicitly or explicitly ask the question, ‘How should one think, judge, and act–as author, narrator, character, or audience–for the greater good?’” (Phelan, 2014, p. 531). The ethical decisions and reflections that fictional characters make are often depicted as situational, and are based on a world-view beyond the binary good or bad, or right versus wrong actions and behaviors. These narrative structures show that decision-making is never simple but is always affected by context, enabling the thick scenarios that are a spur to making moral judgments (cf. Carroll, 1998; Reijers & Coeckelbergh, 2020). This importance of multiple perspectives, engaging content, and complex situations that we find in narratives is also becoming visible within discussions on case studies for teaching ethics [e.g. Herreid (2007), Kim et al. (2006)]. And one way to look at fictional films featuring ethical dilemmas is that they are, in effect, multimodal case studies.

Some of the most popular and engaging fictional films feature narratives that involve speculation on future or alternate worlds. From fables featuring animals that are meant to teach a lesson to the ongoing sagas of superheroes in the Marvel Universe, speculative fictional narratives feature content that nevertheless emerges from and reflects the real world: relationships, choices, individual and community life. They demonstrate possibility as well as potential peril. Pease (2009) explains why these sorts of narratives can help break down barriers to entry for students who are learning about ethics for the first time, and argues that science fiction enables an interdisciplinary investigation of technology and its implications. As such, films focusing on these themes can open the door to ethical considerations that may otherwise have seemed boring or pedantic. Indeed, within engineering ethics education there is an emerging discussion about the use of speculative science fiction for teaching engineering ethics specifically, with positive effects on learning such as enabling practice in deliberation, dialogue, and decision-making, student engagement, engaging diverse learning preferences, and accommodating diverse student backgrounds (Burgess, 2019; Cambra-Badii et al., 2020, 2021; Summet & Bates, 2020). It is logical to assume that fictional films following in the heritage of speculative fiction, whether sci fi, fantasy, or even historical reimaginings or retellings, can do the same.

But film is an effective teaching tool because it is more than a narrative: it is also an emotional, experiential, and aesthetic experience. As a medium it links the narrative possibilities of literature with visual and sonic features to be more immersive. Sinnerbrink (2019) argues that movies are audiovisual spectacles that elicit forms of immersive sensorial experience that captivate our attention at both conscious and unconscious levels of experience (p. 189). Because of this immersion, it is likely that students get a more immediate feel for the different characters, their contexts, and the ethical problems they find themselves in. While film does not put the viewer virtually in the body or experience of another, it does allow the viewer’s imagination to put them there. This creates what Carroll (1998) calls a shared “emotional and cognitive stock” between the film creator and consumer (p. 148). We therefore argue that it is possible for students to understand and feel the moral content of film since it allows the viewer to identify and disidentify with characters and situations (which sutures the viewer to the film), and with the moral content.

Viewers will, as Sinnerbrink (2019) argues, be subjected to an ethical experience when watching a film. However, what is also important to note is that this experience is personal. The connection between the film and the viewer is unique to every person, depending on their experiences, worldview, and personality. Although the different students’ reactions to the film are personal, in some way or another it is the same film that all viewers experience, which creates a common ground for fertile discussions. For instance, Nussbaum (1990) argues that narratives “serve as models of moral reflection and deliberation” as well as provide “occasions for moral understanding” (qtd. in Carroll, p. 148). The different reactions and reflections that students have can also show that there are different ways to view a situation both concerning facts—what happened really? is there some information missing about the situation?—and values—how should we evaluate this situation or the actions of the character? what would I have done if faced with the same situation? Therefore, such personal experiences show that there are different ways to view a film and the actions of the fictional characters, laying the groundwork for intersubjective moral discussions between different viewers of the film and undermining the validity of one single perspective (e.g. what would I do, do I agree, what if the ending were different, etc.).

To illustrate what we have just argued, we show the potential for using film in engineering ethics education in the next section with a discussion of *The Wind Rises*, a 2013 film by Miyazaki Hayao, famous for films such as *Spirited Away*, *Ponyo*, and *My Neighbor Totoro*.

## Teaching Engineering Ethics with *The Wind Rises*

A fictionalized biography of airplane designer Jiro Hirokoshi, *The Wind Rises* explores the personal and professional motivations of a creative boy who found himself working to innovate more efficient and effective Mitsubishi fighter planes in the 1930s and 1940s. Besides being one of the few films that situate an engineer as the main protagonist, it also highlights realistic aspects of engineering practice and deals with a variety of topics relevant to engineering ethics ranging from macroethical concerns to individual decision-making, from aesthetics to cost–benefit analysis. These topics, along with a rich narrative and compelling visuals, make it thick and therefore ideal for investigating ethical issues in the classroom, at a range of breadths and depths.

On a macroethical level, *The Wind Rises* enables viewers to reflect on the connections between engineering and human values. The basic scenario–an engineer improving the capability of a fighting machine in the leadup to war–elicits consideration of the social and political dimensions of engineering. We can see this concept develop most clearly through the character of Jiro’s best friend Honjo, who “constantly reminds him of the real-life socio-political situation in Japan, of Japan’s economic and technological inferiority vis-a-vis the Western powers, and of how Japan is becoming globally isolated and is on a path to destruction” (Daliot-Bul, 2017, p. 568). Thus, while Miyazaki himself believed that “engineers are neutral,” (qtd. in Breen, 2016, p. 457). Breen (2016) argues that *The Wind Rises* enables viewers to explore the “perennial question of the neutrality of technology” (p. 457). This question can be further developed by considering whether an engineer who is fixated on creating beautiful flying machines is wilfully abnegating his social responsibility. In one of Jiro’s dream sequences, the Italian aircraft engineer Giovanni Caproni asks which Jiro would choose: a world with pyramids or one without. There are a number of different interpretations of the scene, but one possible interpretation is that it is about wanting a technology-intensive world, despite the risks and sacrifices that technology entails. Caproni says: “Anyway, I choose a world of pyramids. Which world do you choose?” Jiro replies, “I just want to build beautiful planes.” One implication of this exchange is the reality that engineers and others living in a technology-intensive world cannot simply focus on the beauty of technology, and must also understand its risks and impacts.

Of course, with the benefit of historical hindsight, viewers know all along that Jiro will ultimately design the Zero planes that enabled so much death and destruction. But as the film opens with a scene revealing his childhood dreams of flying, we also know that he does not know the future: this highly creative and motivated boy cannot imagine that his work will ultimately facilitate events like the attack on Pearl Harbor or kamikaze incidents. This perspective encourages viewers to reflect on their own childhood dreams and to consider how their life and work aligns with or departs from those. This kind of perspective-taking exercise has been shown by Burgess (2019) to support and encourage ethical practice in engineering classrooms. Indeed, the film’s perspective on Jiro’s ambitions allow viewers to cheer for his personal success even though they are aware of what is to come, enabling the possibility to consider dual use and the ethical implications of technological development. Jiro declares that he wants his planes to carry passengers, not bombs, and he is often depicted as being obsessed with overcoming a technical problem. Relevant to this, Breen (2016) asks, “are designers complicit in the uses of their inventions?” (p. 458).

Daliot-Bul (2017) agrees that *The Wind Rises* “offers a philosophical investigation into the human tendency to go along and therefore collaborate with peer groups, social norms and authority without questioning the aims” (p. 563). In this way *The Wind Rises* can also serve as a corollary to contemporary events, allowing students to consider their own personal ethical limits (for instance, would I work on military or nuclear technology?) as well as individuals’ responsibilities within corporate ethics. In one episode, Jiro discusses a new aircraft design with his colleagues. The plane is going too slow so they have to make it lose weight. Jiro suggests that “one solution may be… we can remove the weapons!” This is followed by a long silence, and then everyone bursts out laughing. The interpretation of this scene is not straightforward. Does Jiro state this just to produce laughter, or is he trying to voice his concerns and advocate for a peaceful use of aircraft? Asking questions like these allows educators to challenge students’ glib assertions that of course they would “do the right thing” in a situation similar to Jiro’s. Educators can also demonstrate that while a world war is certainly an extraordinary time, so too is our own time of pandemics, war, migration, and severe climate change. In this larger context, *The Wind Rises* asks viewers to consider how personal ambition and visions about the possibilities of technology are affected and influenced by global challenges.

Besides the opportunity to explore the themes of the broader contexts of engineering, ethics educators will find there are many micro-ethical situations that can be analyzed in *The Wind Rises*. These range from the personal to the professional, and often the intersection between the two. As we have described, for most of the film Jiro seems somewhat oblivious to Japan’s growing military-industrial aims. It is when a pilot is killed while testing a new aircraft prototype that Jiro begins to understand the implications of his work. This is such a troubling event that Jiro goes to a mountain retreat in order to recover. There, he can take a step back from his engineering work and reflect upon life and his role as an engineer in the troublesome war situation, and indeed upon his role in the death of the test pilot. During this retreat he meets Castorp, a German who is critical of the Nazi regime and problematizes Japan’s involvement in the War. Furthermore, Jiro meets again with his future wife Naoko Satomi and they fall in love. But also, in the mountains, he builds paper airplanes and in doing so perfects his airplane design. At the end of this sequence Castorp says that this is the magic mountain that cures everyone, and that Jiro who in the beginning of his stay was very gloomy has now been healed and is also very happy because he fell in love. This sequence reveals the way that the whole personhood of an engineer can be affected by issues at work and that love and a temporary distancing could contribute to the work.

*The Wind Rises* also enables the engineering ethics educator to introduce or develop understanding around fundamental ethical theories and questions. For instance, there are many situations within the film that can be analyzed or interpreted through deontological ethics. While some of these are related to Jiro’s professional life, such as his duty to clients, many are also related to his personal life. Jiro’s wife Naoko suffers from tuberculosis, and as she becomes more seriously ill, the question arises whether or not Jiro’s duty to his wife is more important than his duty to his employer. Viewers feel emotionally connected to both Jiro and Naoko, empathizing with her physical deterioration as well as with Jiro’s drive to succeed. This emotional reaction elicits the realization that engineering ethics is never just a simple calculation of risks and benefits, prompting an exploration and critique of utilitarianism. Other elements of the film are suited to an exploration of virtue ethics, like Jiro’s perception of the Italian airplane designer Giovanni Caproni as a moral exemplar, or Jiro’s altruism towards strangers at risk to himself. Too, all of these scenarios can be analyzed through the ethics of care, which privileges emotion and relationships as a path into understanding the responsibilities we have in meeting the needs of others. From a Confucian perspective, one could discuss how Jiro is self-cultivating in pursuit of the human Dao (Way), but also if and how he is an exemplary person—a *junzi*—who also helps other people’s self-cultivation. His relationships with others (such as to his wife, to Honjo, to Caproni, and to his boss), a subset of the five paradigmatic relationships in Confucianism, could be discussed and evaluated based on whether they manifest harmony (Wong, 2012).

Ultimately, the repeated phrase “we must try to live” throughout the film emphasizes that *The Wind Rises* asks us to consider how to live a good life. Even a workaholic like Jiro is affected by (and affects) the world beyond engineering—as a son and husband, as a member of a community, and as a citizen of a nation. Perhaps one of the most important messages that *The Wind Rises* can give to young people studying engineering and who are, like Jiro, often highly motivated, ambitious, and dazzled by technical dreams, is that engineering is only one piece of the mosaic of life.

*The Wind Rises* is a powerful example of the potential that film has for teaching engineering ethics. It follows a narrative structure, and provides a thick presentation of contexts and relationships, in which ethical judgment and decision-making are situated. *The Wind Rises* is also an emotional, experiential, and aesthetic experience. The Studio Ghibli films have a particular aesthetic that “can enrapture and delight everyone from toddlers to pensioners” (Odell & Le Blanc, 2019, p. 1). It is therefore likely that watching the film will create an aesthetic experience for students, akin to the one we have experienced when watching the film. Such experiences are spurs to personal reflection, but the shared experience of aesthetic immersion also creates a fertile ground for discussions and exchanges after the film.

## Discussion

Up until now, we have made the case for using film in engineering ethics education and showcased the potential of the film *The Wind Rises*. Merely watching the film can contribute to a number of learning outcomes, most particularly stimulating moral imagination and sensitivity. In combination with classroom activity, the use of film can also contribute to students’ ethical judgments as well as knowledge about relevant ethical standards. The personal, immersive, and potentially transformative experience that film can provide answers the call for alternative forms of learning in engineering ethics education. Watching film can stimulate the connection “to the heart” which can lead to not only ethical will-power, action, and commitment (Walling, 2015) but also to ethical reflection that is not detached from the life of the person. This connection between engineering ethics and the life of the person, which Mitcham (2009) and Snieder and Zhu (2020) also point out, is experienced via film. The use of film in engineering ethics education can therefore serve as a powerful reminder that the agreed-upon engineering ethics learning outcomes might be too narrowly constructed. In line with Walling, Mitcham, and Snieder and Zhu, we argue that a learning outcome which connects engineering practice to the lifeworld is of great importance in order to emphasize that students also need to be prepared to face ethical issues and situations that resist compartmentalization, spanning a range of spheres (personal, professional, civic) in the life of the person. Such an explicit connection to the lifeworld can also contribute to making engineering ethics education become more appealing, inspiring, and relevant, and avoiding the situation faced by many educators that engineering ethics is seen as a chore. However, educators need to be able to contextualize films used in the classroom and connect them to intended outcomes through teaching methods that make these relationships and purposes explicit.

Facilitating discussion between students is not something that always comes naturally to educators. Therefore, when teaching engineering ethics with film, it is important to create a space for students to reflect on the film, through questions, open small-group discussions, or exercises where the film is scaffolded to theory, codes of conduct, or related ethical dilemmas. Furthermore, given the immersive medium of film, there might be aspects of the film that the teacher considers to be necessary to bring up in a general reflection after viewing the film. Here, an interesting how-to guide is Teays (2017), which suggests five strategies for using film in ethics classes, ranging from open ethical explorations of a film, to exemplifying a theory, a code, or a case using a film (see also Shaw, 2012). Teays’s paper includes a significant number of examples and exercises that the ethics instructor can adopt and be inspired by. However, it lacks explicit links to engineering education and learning outcomes. Hitt and Lennerfors (2021) have described several potential approaches for using film in teaching engineering ethics. They also provide some other avenues for the use of film in class which go beyond watching and discussing film: students can for example produce their own film of an ethical dilemma, rewrite an ending to a film, or similar. These sources provide some suggestions, but do not address limitations or provide empirical data, so more research is needed to develop these approaches. Some limitations that we have encountered through our own experiences as well as in informal discussions with other teachers is that the used film is not appreciated by the students, which leads to skepticism and criticism rather than a productive experience, that some films become rapidly outdated and need frequent replacement, and that the reflection about the film becomes hampered since not enough time and effort is dedicated to adequate classroom activities after watching the film.

In our discussion about the potential of film, we can see that some of these are shared with other pedagogical methods in engineering ethics education. For example, the narrative dimension is shared with fiction as well as with some case studies. The experiential and aesthetic experience is more direct in film than in fiction, where the students’ own imagination is needed to a greater extent. This can make film a more approachable medium, which is appreciated by many students. However, now, with the existence of even more immersive forms of media, such as games and virtual/mixed/augmented reality (also known as XR), the potential for embodied and experiential learning in engineering ethics has been multiplied. Slater et al. (2020) outline the emotional, cognitive, and behavioral effects of XR. They present research showing that the more realistic the sensory rendering in an XR scenario is, the more the participant is affected, and consequently, they argue, the more of an ethical consideration it carries. It remains to be seen how such technologies will be integrated into engineering ethics teaching, and the inclusion of such technologies might spur a range of new ethical issues in the teaching situation (Slater et al. 2020). Perhaps film can for now occupy a suitable middle ground, which is sufficiently immersive, yet safe.

Popular media such as gaming, XR and fictional film can play another important role in ethics education which extends beyond the engineering classroom: they can open up a public conversation about engineering and emerging technologies. When millions of people have seen the same film or streamed the same television series, they have shared in the experience of a narrative, the topics it portrays and the questions it raises. In this way, film can be a way to help the public understand more about engineering and its impacts, an effort seen as increasingly important and indeed as part of the responsibility of engineers. Given the potential and influence of film, film can reinforce positive societal change (Quinn et al., 2011; Mazur & Emmers-Sommer, 2003), and potentially bring more diverse people to the profession, and encourage more creativity and innovation in developing socially responsible and responsive technologies, but also promoting established views of technology and reaffirm society’s beliefs (Buhl, 2021). For these reasons, uncovering, interpreting, and analyzing the often hidden role that engineering plays in film narratives can shape the way that the public understands and recognizes the role of engineers and engineering in daily life.

## Conclusions

As we have shown, fictional films can be an effective way of helping engineering students learn more about ethics. Yet, as Buhl (2021) jokes, “When was the last time you saw a film about the struggles of, say, a network engineer?” This remark highlights the issue we have raised that there are not many films besides *The Wind Rises* that feature engineers as protagonists, and it requires us to consider what other films can be used in teaching engineering ethics. By reframing Buhl’s question to “When was the last time you saw a film about the impacts and implications of engineering?” we begin to see many opportunities. As instructors, we have used films like *Star Wars* and *Gattaca*, and series like *Black Mirror* to highlight these themes, and there are many more that could be used. They may not explicitly name engineers and engineering ethics in their characters and content, but they suggest the many ways in which they are intertwined. Once pointed out to an audience and uncovered via discussions and activities in a learning environment, students can begin to see engineering (and engineering ethics) everywhere. Ultimately, we see using film in engineering ethics education as a way to reframe Phelan’s (2014) question that we cited earlier on, and adapting it to: “how should an engineer think, judge, and act—as designer, builder, technical expert, member of a community—for the greater good?” By creating opportunities to interrogate this question, film can not only improve learning outcomes for students studying engineering but also emphasize the extent to which engineering is a human and social endeavor as much as a technical one.
